# The genome of *Dasychira pudibunda* nucleopolyhedrovirus (DapuNPV) reveals novel genetic connection between baculoviruses infecting moths of the *Lymantriidae* family

**DOI:** 10.1186/s12864-015-1963-9

**Published:** 2015-10-08

**Authors:** Martyna Krejmer, Iwona Skrzecz, Bartosz Wasag, Boguslaw Szewczyk, Lukasz Rabalski

**Affiliations:** Department of Recombinant Vaccines, Intercollegiate Faculty of Biotechnology University of Gdansk and Medical University of Gdansk, 80-822 Gdansk, Kladki Str. 24, Poland; Forest Research Institute, Department of Forest Protection, 05-090 Raszyn Braci Lesnej Str. 3, Sekocin Stary, Poland; Department of Biology and Genetics, Medical University of Gdansk, 80-211 Gdansk, Debinki Str. 1, Poland

**Keywords:** Baculovirus, Next generation sequencing, DapuNPV, Pale tussock moth, *Dasychira pudibunda*, OpMNPV

## Abstract

**Background:**

DapuNPV (*Dasychira pudibunda* nucleopolyhedrovirus), presented in this report, belongs to *Alphabaculovirus* group Ib. Its full, newly sequenced genome shows close relationship to baculovirus OpMNPV isolated from douglas-fir tussock moth *Orgyia pseudotsugata*. Baculovirus DapuNPV is a natural limiter of pale tussock moth *Dasychira pudibunda* L. (syn. *Calliteara pudibunda* L.)(Lepidoptera, *Lymantriidae*), which can occur in a form of an outbreak on many species of deciduous trees and may cause significant economic losses in the forests.

**Methods:**

Late instars dead larvae of pale tussock moth were mechanically homogenized and polyhedra were purified during series of ultracentrifugation. Viral DNA was extarcted and sequenced using Miseq Illumina platform. 294,902 paired reads were used for de novo assembling. Genome annotation, multiple allingment to others baculoviruses and phylogegentic analises were perform with the use of multiple bioinformatic tools like: Glimmer3, HMMER web server, Geneious 7 and MEGA6.

**Results:**

The genome of DapuNPV is 136,761 bp long with AT pairs content 45.6 %. The predicted number of encoded putative open reading frames (ORFs) is 161 and six of them demonstrate low or no homology to ORFs previously found in baculoviruses. DapuNPV genome shows very high similarity to OpMNPV in a nucleotide sequence (91.1 % of identity) and gene content (150 homologous ORFs), though some major differences (e.g. lack of he65 in OpMNPV) have also been noted.

**Conclusions:**

Similarly to other members of the *Baculoviridae* family, DapuNPV baculovirus possesses highly conserved core genes. Among them, there is a second copy of occluded derived virus envelope 27 protein (*odv-e27*), which was previously found only in a member of *Alphabaculovirus* group II – LyxyMNPV (*Lymantria xylina* MNPV). Surprisingly enough, DapuNPV and LyxyMNPV genomes share also another feature. Phylogenetic analysis of chitin binding family protein (*cbpl*) indicates significant similarity of those two baculoviruses from distinct evolutionary groups which infect the same hosts from *Lymantriidae*. The ubiquitin like family gene (*ubil*), which has not been described until now, is another characteristic component of DapuNPV genome.

**Electronic supplementary material:**

The online version of this article (doi:10.1186/s12864-015-1963-9) contains supplementary material, which is available to authorized users.

## Background

The *Baculoviridae* family is a major group of viruses infecting insects. Its members are enveloped, rod-shaped viruses with covalently closed, circular, double-stranded DNA [1, 2]. The genome size ranges between 81,755 kbp (NeleNPV, *Neodiprion lecontei* nucleopolyhedrovirus [3]) and 178,733 bp (XcGV, *Xestia c-nigrum* granulovirus [4]). It has been reported that the *Baculoviridae* family contains 37 core genes [5].

Based on morphology, *Baculoviridae* family was originally subdivided into two genera: nucleopolyhedroviruses (NPV) forming polyhedral occlusion bodies (OBs) with single or multiple virions inside, and granuloviruses (GV) with single virions inside ovoid OBs made of granulin [6–8]. In 2006, based on genetic sequences and due to host-associated virus evolution new classification was proposed. According to this nomenclature, the family was divided into: *Alphabaculovirus* (lepidopteran-specific NPVs), *Betabaculovirus* (lepidopteran-specific GVs), *Gammabaculovirus* (hymenopteran-specific NPVs) and *Deltabaculovirus* (dipteran-specific baculoviruses with only one representative *Culex nigripalpus* nucleopolyhedrovirus (CuniNPV)) [8–10]. *Alphabaculovirus* genus consists of group I and II, depending on the presence or absence of BVs envelope glycoprotein gp64. Only the members of group I contain gp64, while gene encoding fusion protein (F) indicates affiliation to group II (as well as *Beta-* and *Deltabaculoviruses*) [11–14]. It is suggested that group I originated from ancestral group II of *Alphabaculovirus* [15].

During the life cycle of the virus two different virion phenotypes are observed: occlusion derived virus (ODV) and budded virions (BV). The first one is responsible for spreading infection between caterpillars, while the second one for cell-to-cell propagation inside a larvae [1, 2]. The members of all *Baculoviridae* family produce ODV, while BV is produced only by three genera: *Alpha-, Beta*- and *Deltabaculovirus* [16].

The pale tussock moth *Dasychira pudibunda* L. (syn. *Calliteara pudibunda* L.) (Lepidoptera, *Lymantriidae*) is a frequent defoliator with economic significance. Larvae prefer mainly deciduous trees, especially *Fagus spp*., but they can feed on many different plant species like: *Quercus, Betula, Corylus, Tilia, Juglans, Salix, Populus*, fruit trees and shrubs in orchards as well; very rarely on conifers [17, 18]. This pest is present in the entire Europe except for Far North, but causes outbreaks only in the area of western and central part of the continent (between 48th and 57th parallels) [19]. Since 1810, massive infestations have been reported mainly in Germany [20, 21], Sweden [22–24] and Denmark [25] – all on *Fagus* trees. In central-eastern Europe in countries such as Poland, Romania and Ukraine, the main host plant during outbreaks was *Quercus robur* L. [21, 26]. *Dasychira pudibunda* L. occurs also in central Asia, China and Japan. Infected larvae used in these studies were collected in 2010 in Northern part of Poland from 50-year *Fagus sylvatica* L. trees growing in the State Forest. At present, this pest is relatively rare in Poland and does not cause problems in forests [27].

One of the agent that is able to control mass occurrence of the pale tussock larvae is a baculovirus. Viral disease which often cause epizootic are responsible for long protection of trees against reestablishment of a pest, therefore the baculovirus is an environmental friendly biopesticide [28]. Although the existence of *Dasychira pudibunda* nucleopolyhedrovirus (DapuNPV) has been occasionally reported in the literature [29], so far there is no genome sequence available in the National Center for Biotechnology Information (NCBI) database. Here, we report the first full genome sequence of this baculovirus, that shows its close relation to *Orgyia pseudotsugata* MNPV (OpMNPV), *Choristoneura murinana* MNPV (ChmuMNPV), *Choristoneura rosaceana* MNPV (ChroMNPV) and *Choristoneura fumiferana* MNPV (CfMNPV).

## Results and discussion

### General characteristics of the DapuNPV genome

**Fig. 1:**
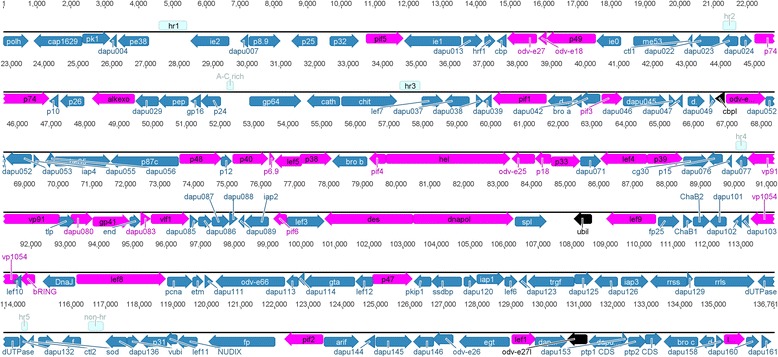
Linear map of the DapuNPV genome. Arrows represent orientation of predicted ORFs; different groups of annotated ORFs are colored: pink – baculovirus core genes, blue – all other DapuNPV ORFs, black – genes of special interest: *cbpl*, *ubil* and *odv-e27l*; light blue rectangles represent repeat regions

The complete circular DapuNPV genome is 136,761 base pairs long (Fig. 1). It is 4,766 bp longer than the genome of the most closely related baculovirus OpMNPV. Between DapuNPV and OpMNPV there is 91.1 % similarity in nucleotide sequence based on whole genome MAFT alignment. The AT pairs content is 45.6 % and classifies DapuNPV in the vicinity of OpMNPV with its 44.9 %, while the highest AT pairs content among the members of *Baculoviridae* family is found in CrleGV (*Cryptophlebia leucotreta* granulovirus) - 67.6 % and the lowest in LdMNPV - 42.5 %. Intergenic regions of DapuNPV consist of 12,853 bp (9.4 % of whole genome) with AT pairs content calculated to be 59.8 %.

The number of putative open reading frames (ORFs) in DapuNPV is equal to 161, nine more than in was annotated in OpMNPV genome (Additional file 1: Table S1). Two homologues to ORFs: *dapu077* and *dapu132* were not described in OpMNPV due to substitutions associated with changes in stop codons. From all DapuNPV ORFs found during homologue search using hidden Markov models analysis (HMMER) (see Methods) three had no hits (*dapu024, dapu047, dapu055*) and another three had E value higher than 0.01 (*dapu046, dapu048, dapu132*), which was treated as insignificant. Despite the fact that OpMNPV genome was annotated in the manner similar to AcMNPV, we chose to designate *polh* (gene encoding for polyhedrin) as first ORF in clockwise orientation, as it is adopted in recent full baculovirus genomes reports. According to this criterion, there is 76 ORFs in forward orientation and 85 in reverse orientation in DapuNPV genome. The ratio of forward to reverse located genes is almost the same in OpMNPV (72:80) and distinct from CfMNPV (73:73) and HycuNPV (*Hyphantria cunea* nucleopolyhedrovirus) (69:79).

During NGS coverage analysis (i.e. how many single reads can be mapped to a corresponding location in the genome, which allows for the detection of possible recombinant viruses in a population) only a small narrowing can be observed in intergenic regions. The most significant one appears in *A-C rich* region because of high di-nucleotide sequence composition. To confirm correct *de novo* assembly, the standard Sanger’s sequencing was performed for this region. We detected one site of Single Nucleotide Polymorphism (SNP) that is located at the position 542 from beginning of *p32* gene. This G to A transition change translated amino acid from Arginine to Glutamine.

Recently discovered in *Alphabaculovirus* genus, a conserved, 156 bp long, non-protein-coding genomic element (CNE), that is presumed to be responsible for virus replication in transfected insect cell cultures, is present also in the DapuNPV genome [30]. The CNE is located from 6,994 bp to 7,149 bp and partially overlaps with *dapu007* ORF. This non-coding region shows the highest sequence identity (98.1 %) and similar sequence composition (AT content 60.9 %) with the corresponding one of OpMNPV.

**Fig. 2:**
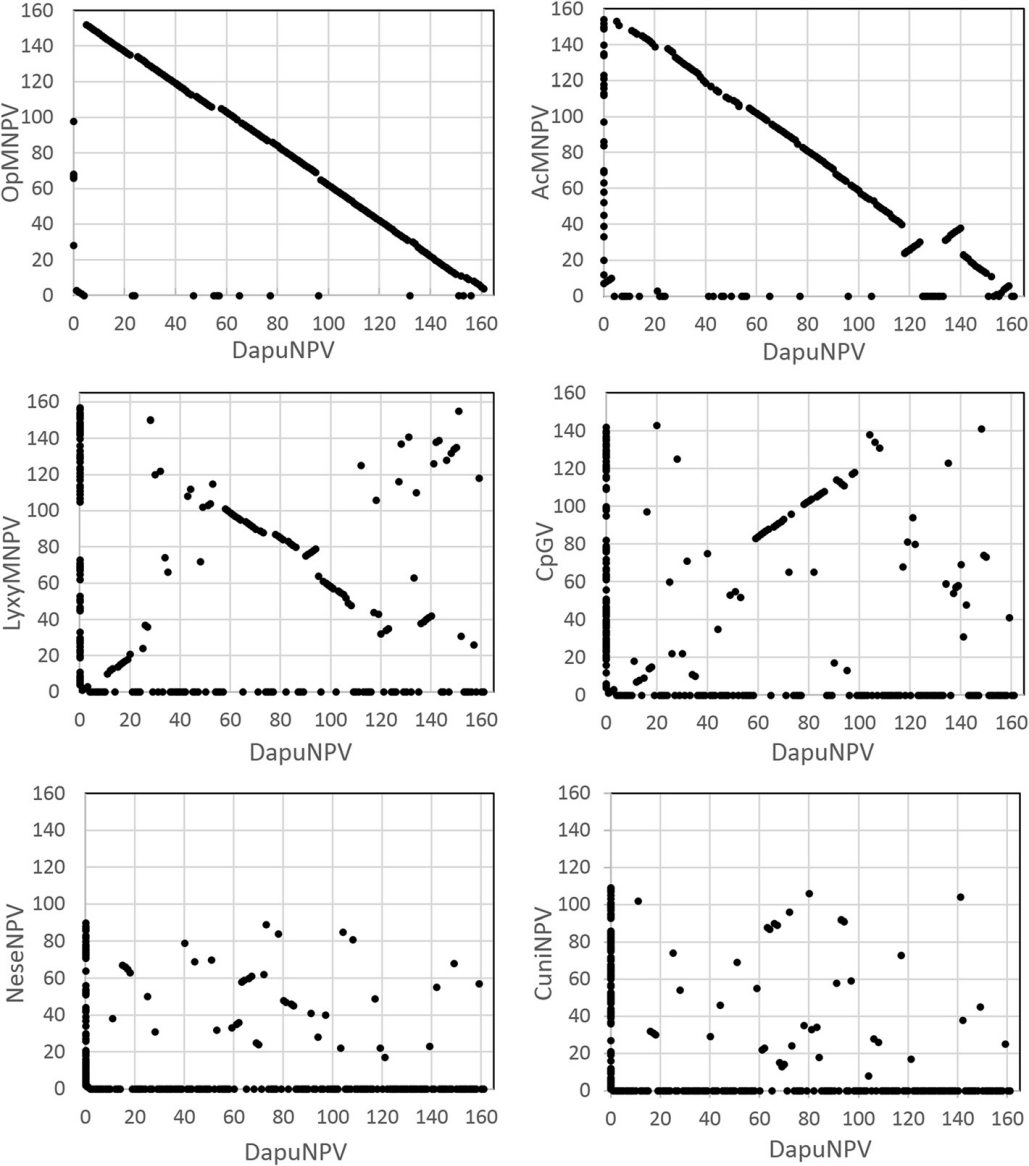
Gene-parity plot analysis. Gene-parity plots of DapuNPV with OpMNPV, AcMNPV, LyxyNPV, CpGV, NeseNPV and CuniNPV based on ORF order where *polh* gene is considered as a first gene

**Fig. 3:**
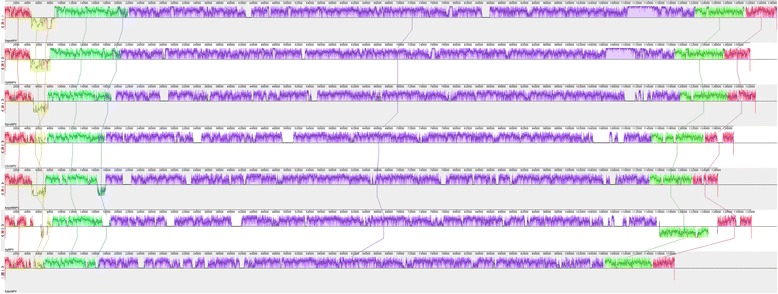
Mauve (multiple alignment of conserved genomic sequence with rearrangements) representation of *Alphabaculoviruses* from group Ib. Alignment was performed on collinear sequences where DapuNPV was a reference sequence and *polh* gene was considered as a first ORF. Colored sections (bordered with a curve that indicates the level of nucleotide similarity) represent homologous fragments of compared genomes. Section that is located beneath the X-axis shows inversion of this genome fragment in comparison to the reference. Genomes presented DapuNPV, OpMNPV, HycuNPV, ChroNPV, AnpeNPV, AgNPV and EppoNPV

Gene-parity plot analysis of DapuNPV against representatives from *Alphabaculovirus* Ia – AcMNPV (*Autographa californica* MNPV), *Alphabaculovirus* Ib - OpMNPV, *Alphabaculovirus* II – LyxyMNPV (*Lymantria xylina* MNPV), *Betabaculovirus* – CpGV (*Cydia pomonella* granulovirus), *Gammabaculovirus* – NeseNPV (*Neodiprion sertifer* NPV) and *Deltabaculovirus* – CuniNPV is shown in Fig. 2. Graphical interpretation of homologous blocks in viral genomes from group Ib is presented in Fig. 3. Types of recombination such as inversions and dislocations were detected between DapuNPV ORFs: *pe35-pif5* (for Choristoneura spp*.* NPVs, AgNPV (*Anticarsia gemmatalis* NPV) and EppoNPV (*Epiphyas postvittana* NPV), *me54-p74* (for AnpeMNPV -*Antheraea pernyi* NPV-), PhcyNPV (*Philosamia cynthia* ricini NPV) and *F-protein-dapu152* (for AgNPV, CfDEFMNPV (*Choristoneura fumiferana* defective MNPV)). The gene encoding superoxide dismutase *(sod*) was also inversed in AgNPV, CfDEFMNPV, PhcyNPV, AnpeMNPV and HycuNPV genomes.

### Phylogenetic relations to other baculoviruses

**Fig. 4:**
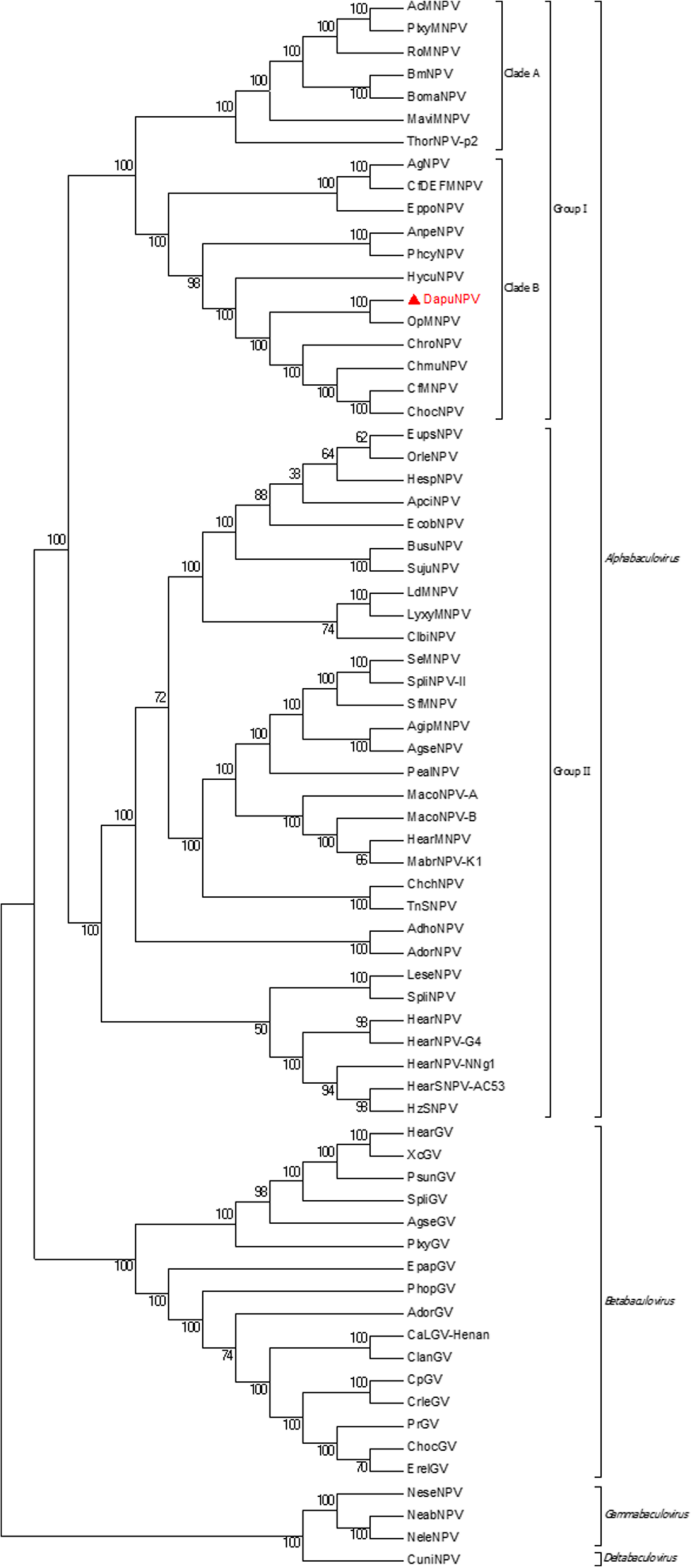
Maximum Likelihood molecular phylogenetic analysis of 70 baculoviruses. Amino acid sequences of 37 core genes were analysed. The position of DapuNPV (labeled with red triangle) was shown clustering with OpMNPV in *Alphabaculovirus* group Ib

**Fig. 5:**
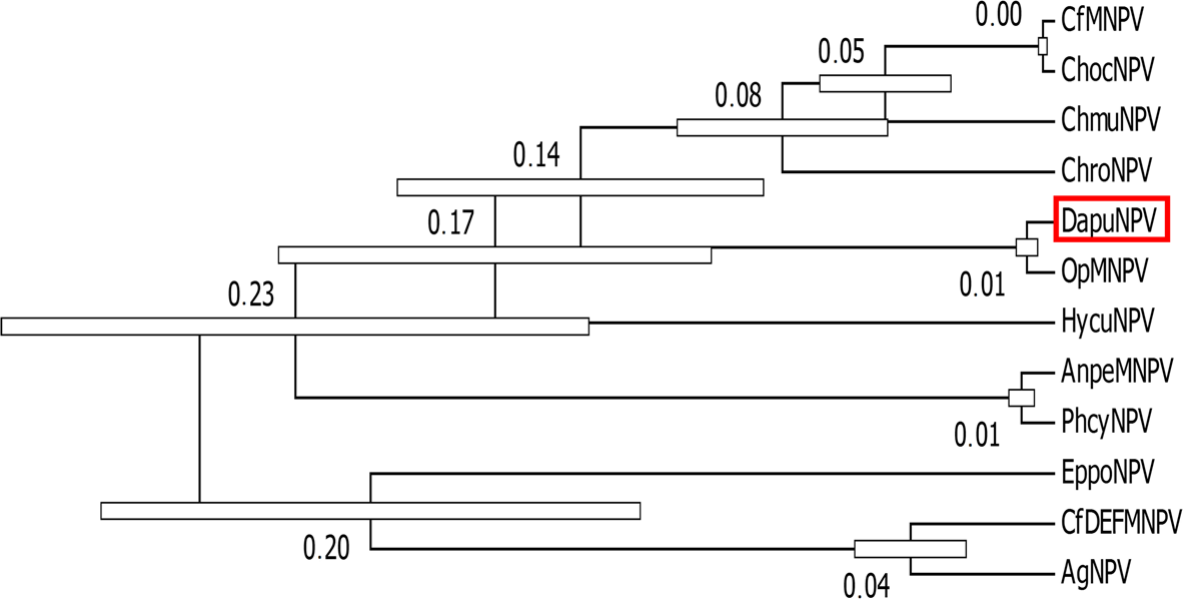
Time tree molecular phylogenetic analysis of *Alphabaculovirus* group Ib. Collinear homologue nucleotide regions of baculoviruses were analysed for their possible evolution history. The approximate relative time of divergence between two viruses is visualized by the wide bar on tree node. DapuNPV is highlighted by a red rectangle

According to its gene content, including *gp64* and *F-protein,* DapuNPV is classified as a typical representative of the *Alphabaculovirus* group Ib [31]. To confirm this classification, phylogenetic Maximum Likelihood tree presented on Fig. 4 was generated using the alignments of each of the 37 single core genes concatenated into one MAFFT alignment (multiple sequence alignment based on fast Fourier transform). All 69 baculovirus genomes available in NCBI database until December 2014 and DapuNPV from this report were used (Additional file 2: Table S2). The nearest location of DapuNPV to OpMNPV on the phylogenetic tree is an additional confirmation of coevolution events between viruses and their hosts [9]. Both viruses were isolated from infected *Lymantriidae* caterpillars. To affirm this relationship, we conducted evolutionary analyses by calculating relative divergence times for all fully sequenced baculoviruses group Ib using nucleotide sites within the scope of collinear genes. The “RelTime” method used for creating tree presented in Fig. 5 indicates that possible divergence of ancestor baculovirus to DapuNPV and OpMNPV might have happened in the recent past compared to the first divergence in the clade. The method used for this deduction relies on the previous inferred molecular evolution and does not need any calibration events (e.g. absolute dating based on fossils). In a time span analogous for the divergence of DapuNPV and OpMNPV, two other pairs of closely related baculoviruses (AnpeMNPV and PhcyNPV, CfMNPV and ChocNPV) underwent similar evolutionary episode. The concurrence of these events may indicate occurrence of a particular timing when baculoviruses underwent their adaptation to hosts.

### Repetitive genomic regions

Homologous repeats (*hrs*) are characteristic for almost all baculoviruses sequenced to date and consist of direct repeats, imperfect palindromes and high AT content. Within one viral genome they show homology, but it is hard to find similarities between different baculoviruses, except in those closely related. *Hrs* have been considered as origins of DNA replication in NPVs and GVs and transcriptional enhancers of early genes in NPVs (reviewed in [32, 33]). Additionally, non-homologous region (*non-hr)* origins of replication have been found in some NPVs [34].

**Fig. 6:**
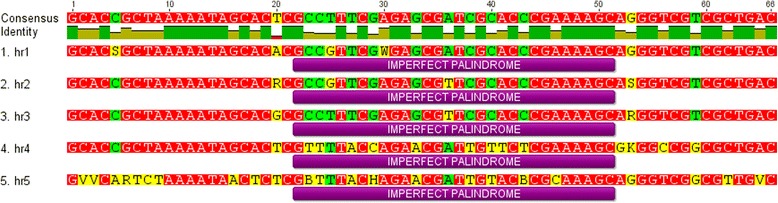
Multiple alignment of all DapuNPV *hr* regions. Bar below sequences highlights palindrome core region (MAFT multiple alignment with default settings, gaps ignored, visualization in Geneious R7)

The genome of DapuNPV has three types of repeated nucleotide sequences (Table 1): five homologous regions (from *hr1* to *hr5*), one non-homologous region and one Adenine-Cytosine rich region (*A-C rich*). They all have highly similar equivalents in OpMNPV genome. The sequence and location of homologous regions are often conserved between related baculoviruses, although their lengths and copy numbers can vary from species to species. Every repeated element in DapuNPV *hrs* is composed of 30 bp imperfect palindrome core sequence flanked by regions which are also highly conserved. Figure 6 shows multiple alignment of strict repeated elements consensus from each *hr*.

**Table 1:** Repeated regions of the DapuNPV

Repeat region name	Strict consensus nucleotide sequence	Repeated element length	Repeated element copies^a^	Location ORF
*hr1,*	GCACCGCTAAAAATAGCACDCGCCTTTCGAGAGCGATCGCACCCGAAAAGCAGGGTCGTCGCTGAC	66	13	*pe38 – ie2*
*hr2,*		66	6	*dapu023*
*hr3,*		66	10	*chit – lef7*
*hr4,*		66	5	*dapu077*
*hr5*		66	4	*dapu132*
*non-hr*	AAARTRATGAYTCATGYTADAKCAAGGTY	29	16	*fgf – dapu136*
*A-C rich*	AMACAMACAMAC	12	16	*p24 – gp64*

^a^every truncated element was counted as one

Surprisingly, DapuNPV *hrs* share higher level of sequence and localization similarity with HycuNPV than with Choristoneura spp*.* which is opposite to the genetic distance based on inferred evolutionary history of collinear genomes fragments (Fig. 5)*.* Non-homologous repeated region occurs only in DapuNPV and OpMNPV, but some other *Alphabaculoviruses* group Ib can have homologous repeats in the corresponding sites of genomes (ChroNPV, ChocNPV, CfMNPV and EppoNPV). Another repeated element that is found only in DapuNPV and OpMNPV is an *A-C rich* region. In DapuNPV it has 222 bp, it is around four times shorter than in OpMNPV baculovirus.

One of the characteristic features of the genomic sequence that flanks *hr* regions is a high level of rearrangements (inversions, dislocations, deletions and insertions). These genome variations occur even between closely related baculovirus species. For example, conotoxins-like (*ctl*) proteins are found in approximately one third of baculoviruses sequenced to date and some of them possess two genes of different lineages – *ctl-1* and *ctl-2*. Conotoxins are a big family of small disulphide-bonded peptides, ion channel antagonists firstly isolated from marine cone snail venom. They are classified based on their disulphide pattern and biological features [35]. In baculoviruses *ctl* is transcribed as a late gene and its product is a small secretory peptide not involved in viral infection *in vivo* or *in vitro* [36]. Multilocus presence of *ctl-1* and *ctl-2* occurs frequently in all *Alphabaculovirus* group Ib. In DapuNPV *ctl-2* is located after *hr5* in the counter-clock orientation. Its homologue gene is inversed in HycuNPV, whilst entirely deleted in ChocNPV and AnpeMNPV.

### Two chitin binding protein families

**Fig. 7:**
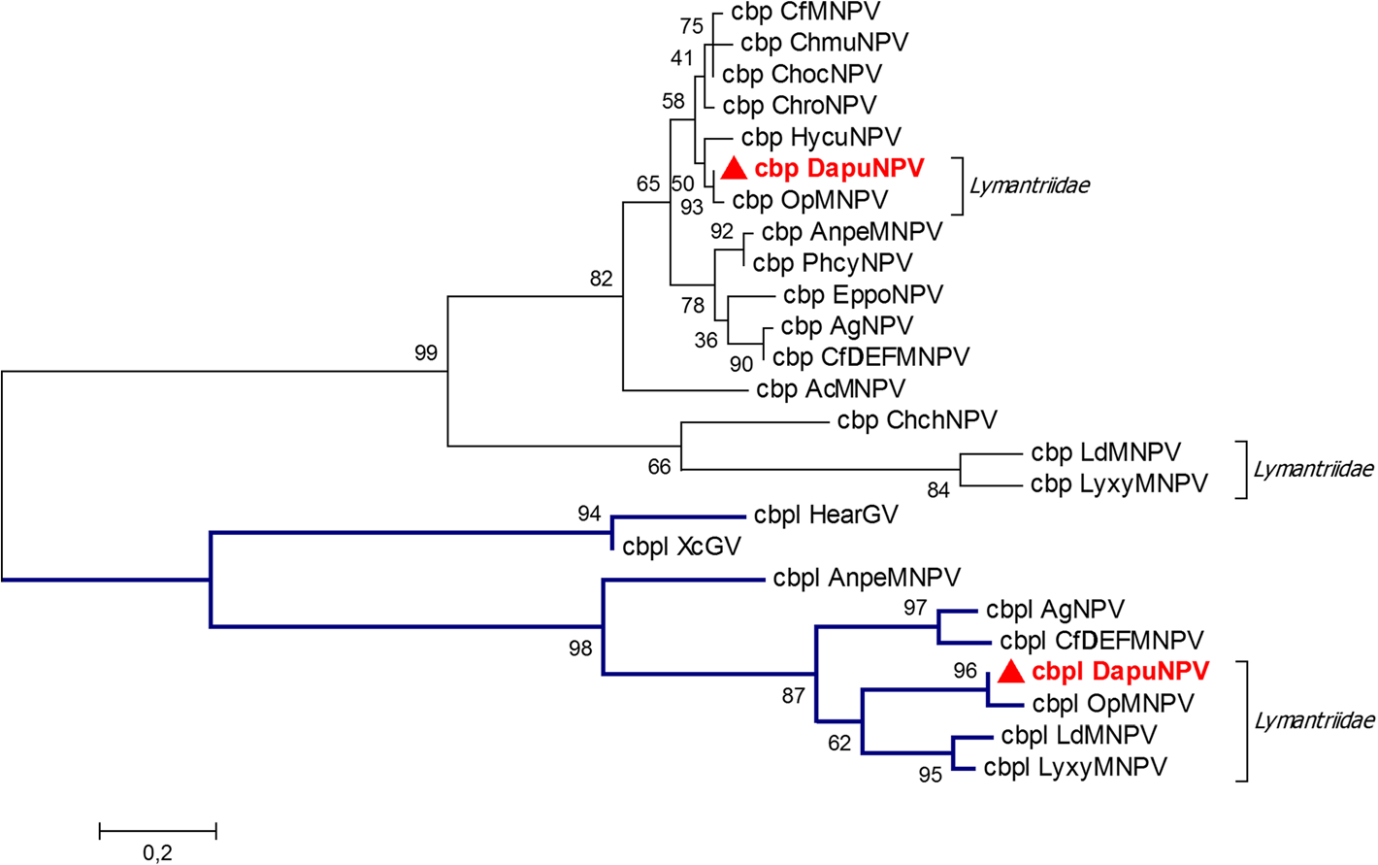
Maximum Likelihood molecular phylogenetic analysis of two chitin binding baculoviruses proteins. Two (*cbp* and *cbpl*) similar genes show different pattern of clustering. *Cbpl* highlighted by blue tree branch groups more host-specific species than *cbp*. The positions of DapuNPV was labeled with a red triangle

Chitin binding protein (*ac145*) is recognized in all groups of baculoviruses except for *Deltabaculoviruses*. It is considered as a possible *pif* gene (*per os* infectivity factor) that plays a significant role during early stages of larvae infection, as its deletion leads to a decrease in pathogenicity [37]. Sequencing of DapuNPV identified two ORFs, *cbp* (*ac145*) and *cbpl* (chitin binding protein like)*,* as homologues of *ac145*. Protein encoded by *cbpl* gene (homologues were previously found in OpMNPV, AgNPV, CfDEFMNPV and AnpeMNPV, but not in other *Alphabaculoviruses* from group I) is composed of a signal peptide with a region similar to arthropod chitin binding peritrophin-A domain. Proteins with this domain are members of type I peritrophic membrane (PM) proteins, and by binding chitin they form a protective coating of the larvae midgut [38, 39]. Surprisingly, HMMER search showed that DapuNPV and OpMNPV *cbpl* genes show higher level of similarity to *Alphabaculoviruses* from group II - LdMNPV and LyxyMNPV than to other, closely related baculoviruses. Unrooted ML tree (Fig. 7) confirmed that baculoviruses infecting insects from different species of *Lymantriidae*, possess *cbpl* genes that are more similar to each other than to *cbp* genes even of the same species. This indicates that despite their structural similarities, *cbp* and *cbpl* do not arise from single gene duplication, but rather by horizontal transfer. In addition, our hypothesis on the origin of these proteins is supported by high bootstrap replicates. Probably, *cbp* and *cbpl* have the same function during viral life cycle. This would suggest that an evolutionary event (horizontal gene transfer) that occurred in the past, was fixed in those baculoviruses that can benefit from an additional factor able to disturb chitin-protein larvae peritrophic membrane rather than from replicon enlargement. Phylogenetic relationship of *cbp* presented on the same tree is consistent with general baculovirus classification (Fig. 7).

### DapuNPV and OpMNPV genomic diversity

We identified a nucleotide sequence that is 97.6 % identical to ORF *dapu004* in OpMNPV, but is has not been annotated there. This gene with late promoter motif and intrinsically unstructured/disordered protein fragment has its homologue in other *Alphabaculoviruses* Ib (*chro004*). Based on its predicted structure, this protein, if transcribed, could potentially contribute to cell-cycle regulation, signal transduction or transcription [40, 41].

Highly basic p8.9 protein was described as an unique 228 bp long ORF in OpMNPV. In DapuNPV genome *p8.9* ORF is built of 942 bp due to the large insertion in its central region (Additional file 3: Figure S1). Search against Pfam database do not yield any results, though the confirmed translation in OpMNPV supports the hypothesis that the homologue of p8.9 protein plays a role in DapuNPV life cycle. The predicted molecular mass for this DapuNPV protein is approximately 36 kDa. The composition of four basic amino acids specific for OpMNPV p8.9 is about 30.2 %, while for DapuNPV it is equal 25 % [42, 43].

A universal trait of many eukaryotic dsDNA viruses is the presence of multigene families (MGFs) containing related repeated open reading frames (ORFs) scattered within the genome. The *Baculoviridae* family also contains their own group of such genes called *bro* genes (baculovirus repeated ORFs), that differ in number and length from one virus to another [44]. These sequences are present in species from all genera, except in *Gammabaculovirus*, in up to 16 (LdMNPV) copies per genome [45]. However, they are absent in RoMNPV (*Rachiplusia ou* MNPV) [46] and PlxyGV (*Plutella xylostella* GV) [47]. DapuNPV genome has three *bro* ORFs (a, b and c). *Bro a* homologues can be found in all *Alphabaculoviruses* Ib; *bro b* as a functional homologue is present in HycuNPV, AnpeMNPV and PhcyNPV, while *bro c* can be recognized in HycuNPV, CfDEFMNPV, AgNPV. In ChocNPV and CfMNPV the latter two *bro* genes are shorter and dislocated. OpMNPV genome encodes a shorter homologue than DapuNPV *bro a* gene. Variations in numbers, locations and content of baculovirus *bro* genes between closely related species are their well-known characteristic [44].

DapuNPV nucleotide sequences corresponding to *dapu023* and *dapu024* are only partly present in OpMNPV genome in *hr5* region. Because in OpMNPV this fragment is much shorter than in DapuNPV it does not form any ORF. DapuNPV consecutive ORFs *dapu046, dapu047* and *dapu048* have no homologues in any dsDNA viruses. An early promoter motif is placed upstream the first gene, whilst the remaining two genes encode transmembrane domain containing protein and a single prokaryotic RING finger family four domain containing protein respectively, which may indicate a bacterial origin of this fragment of the genome. In the same region of OpMNPV genome (between *op111-op114* that corresponds to *dapu045-dapu049*), two conserved ORFs are present but are not found in DapuNPV.

Two ORFs, not previously described, with early promoter motifs (*dapu55* and *dapu56*) are located after DapuNPV *iap4* gene. The nucleotide sequence corresponding to this two genes cannot be found in OpMNPV genome. DapuNPV *iap4* is almost half shorter than OpMNPV *iap4* because of a 88 bp deletion and a frame shift. Subsequent ORF *he65* of DapuNPV shows 73 % nucleotide identity to *he65* from *Alphabaculovirus* Ib AnpeNPV where in OpMNPV only 205 bp of 3’ of full 1650 bp DapuNPV gene is present. This OpMNPV short region does not code putative gene. He65 protein is one of the representatives of RNA ligases family and is prevalent in *Archea*, *Bacteria* and *Eukarya*. It is an early transcribed gene involved in RNA replication, transcription and modification, as well as in G-actin localization in nucleus during AcMNPV-cells infection. It is considered as a nonessential protein for AcMNPV and BmNPV [48, 49]. Homologues of *ac105* encoding *he65* gene are found in 27 baculovirus genomes (sequenced to date; genes called *he65* or annotated as similar to *ac105*). They are present in 22 members of *Alphabaculovirus* genus: in all seven from group Ia, five from Ib (among others *ag101*, *cfDEF*97 and *dapu57*), ten from group II (e.g. *agip25*, c*hch73*, *lese68*) and five members of *Betabaculoviruses* (*agse132*, *erel36*, *hear62*, *psun63*, *xc67*). In almost half of these cases *he65* gene is placed close (upstream or downstream) to late gene encoding structural vp80/p87 protein (NPV-specific protein).

Another ORF that shows INDELS (insertions-deletions) type variation between DapuNPV and OpMNPV is *dapu071,* where the deletion of 228 bp located in the middle of the gene does not change the reading frame.

The region in the sequence between DapuNPV *spl* (spheroidin-like) and *lef9* genes is highly variable in *Alphabaculoviruses* Ib. For example in OpMNPV and ChroNPV there is a *bro* gene, absent in DapuNPV. This dissimilarity is 650 bp in length in OpMNPV and 1394 bp in DapuNPV, where 534 bp encode ubiquitin like protein (*ubil*); its gene is controlled by an early promoter and does not have a homologue in any baculoviruses (the closest search hit during HMMER was a Polyubiquitin-C protein from *Mus musculus;* E value 1.2e-22). Additionally *ubil* gene locus (it does not overlap with other ORF) and protein size (177 amino acids) confirm of its existence. Ubiquitin is highly conserved and abundant in all eukaryotes, which indicates its crucial role in cell cycle. The main function in a cell is to direct proteins to degradation pathway but it also takes part in stress response, ribosome biogenesis, and cell differentiation [50]. DapuNPV genome encodes also another ubiquitin gene (*vubi* - viral ubiquitin) whose homologues have been found in wide range of viruses [51]. Viral ubiquitin proteins are characteristic for all lepidopteran baculovirus species, that make them core genes for *Alpha*- and *Betabaculovirus*, none of them is present in dipteran- or hymenopteran-specific members of *Baculoviridae* family [5]. DapuNPV ubiquitin like (*ubil)* seems not to be related to viral ubiquitin (*vubi)* gene conserved in baculoviruses as it has only 30 % nucleotide identity and 17 % amino acid identity.

### DapuNPV is a first *Alphabaculovirus* group I with two copies of *odv-e27* gene

Odv-e27 is a structural protein. Although it was named odv-ec27 (occlusion derived virus envelope and capsid [52]) it is present in both phenotypic forms of baculoviruses like ODV and BV [53]. It is also presumed to be essential for budded virus production [54]. Odv-ec27 was also reported to act as a viral cyclin that participates in cell cycle regulation during infection [55]. Homologues of gene *odv-ec27* (*ac144*) belong to core genes, so it is prevalent in all sequenced to date baculoviruses [5] in one copy, except for single *Alphabaculovirus* group II - LyxyMNPV where two genes have been identified [56]. The DapuNPV is a second baculovirus with additional copy of *odv-e27* gene. While DapuNPV 194 amino acids (aa) protein is similar to smaller copy of LyxyMNPV protein odv-e27 (188 aa), it shares only 25.1 % BLSM62 pairwise positivity (23 % amino acid identity). Odv-e27 proteins that are encoded by baculovirus core genes are more similar to each other than to DapuNPV odv-e27l (odv-e27 like protein) or shorter LyxyMNPV odv-e27 protein. Inferred evolutionary history for 70 core gene *odv-e27* and two short versions form DapuNPV and LyxyMNPV revealed that shorter versions cluster together distantly from core genes (data not shown). The duplication of *odv-e27* genes and close phylogenetic relationship of *cbpl* homologues reveals a surprising connection between evolutionary distinct *Alphabaculoviruses* that infect insects from the *Lymantriidae* family.

### Protein tyrosine phosphatase

In general, parasites are known to change their hosts behavior during infection. A great diversity of behavioral changes may occur such as in feeding, mating, odor response and activity. In most cases direct molecular causes have not been yet described [57]. However, in baculoviruses, *egt* gene has been noted as responsible for ‘tree top disease’, i.e. it induces climbing behavior in gypsy moth *Lymantria dispar* L. caterpillars during late stages of infection, in order to spread progeny virus on a wider range of healthy larvae [58]. Another gene *ptp* encoding protein tyrosine phosphatase has also been shown to participate in behavioral changes of the host *Bombyx mori* L. by strongly enhancing locomotory response to infection in caterpillars, which are normally almost immotile [59, 60]. The viral ptp is a component of the virion, consists of HC motif, where cysteine residues are necessary for its function as an active phosphatase [61]. Protein tyrosine phosphatase gene is present in all lepidopteran NPVs in group I. Phylogenetic analysis implies that *egt* as well as *ptp* genes appear to have a lepidopteran origin [62]. During the annotation process of the assembled DapuNPV genome, all different tools used predicted three genes that are located one behind the other. We named them *dapu123, ptp1* and *ptp2.* Comparative genomic analyses between DapuNPV and *Alphabaculoviruses* show that *ptp* genes are highly conserved in genomic sequence, but in many species *ptp2* is not present (AcMNPV, HycuNPV, EppoNPV). ORF *dapu123* was not predicted in any baculovirus, mainly due to a mutation that changes a reading frame which introduces STOP codon earlier to predicted transcript (*Choristoneura spp*. viruses). A genome fragment responsible for this three ORFs is in 98 % similar to OpMNPV. However, in OpMNPV baculovirus, *ptp1* was annotated as a longer gene that has its start inside DapuNPV *dapu123* gene. This ORF enlargement in OpMNPV is not supported by genomic sequence from any analyzed *Alphabaculovirus* group I. Since comparative genome data is consistent with the data obtained from the bioinformatic tools that we used, we decided to distinguish ORFs: *dapu123* and *ptp1* (wherein the second one starts from a codon located 129 bp downstream in comparison to its OpMNPV homologue).

## Conclusions

DapuNPV baculovirus is closely related to OpMNPV but possesses a few distinctive features. The most prominent ones are genes similar to *ubi* (called *ubil*) and *odv-e27* (called *odv-e27l*). The first gene does not have any homologue in baculoviruses while the second one can be found only in one member of *Alphabaculovirus* group II – LyxyMNPV. This connection between members of evolutionary distinct groups of baculoviruses which infect the same hosts from the *Lymantriidae* is also supported by the shared homologue *cbpl* gene. DapuNPV and OpMNPV have 91.1 % of nucleotide uniformity though there are some differences in gene content (e.g. *he65*). Study on phylogenetic relationship between baculoviruses was prepared based on alignment containing amino acid core gene sequences from 70 members of *Baculoviridae* family that were manually picked and confirmed.

### Nucleotide sequence accession number

The DapuNPV genome sequence was submitted to GenBank under accession number KP747440.

### Availability of supporting data

The complete DapuNPV genome sequence has been submitted to GenBank (accession number KP747440). All supporting data are included as additional files.

## Methods

### Virus purification and DNA extraction

Late instars of pale tussock moth caterpillars infected by baculovirus were collected in 2010. The presence of polyhedra was confirmed with light microscope. Larvae were mechanically homogenized in deionized distilled water (ddH_2_O). The homogenate was filtered through cheesecloth to remove pieces of cuticle. Afterwards the filtrate was centrifuged at 5000 × g for 10 min in room temperature (RT). The supernatant was discarded, the pellet consisting of polyhedra was resuspended in 0.5 % SDS and centrifuged at 5000 × g for 10 min at RT. Then again the pellet was resuspended in 0.5 M NaCl and centrifuged as before. The pellet was resuspended in ddH_2_O and loaded onto a linear 40–65 % sucrose gradient and ultracentrifuged at 96,000 × g for 3 h at RT. The collected milky polyhedral band was diluted in ddH_2_O and pelleted by centrifugation at 10,000 × g for 10 min at 4 °C (this step was repeated twice). After final resuspension polyhedra were dissolved in an alkaline solution (0.1 M Na_2_CO_3_, pH 10.0) for 30 min. Equal volume of Tris pH 6.4 buffer was used for neutralization followed by DNA extraction by MagAttract HMW DNA Kit (Qiagen) according to manufacturer’s protocol. DNA prior to sequencing procedure was diluted in nuclease-free water.

### Genome sequencing and assembly

DNA sequencing was done at the Medical University of Gdansk in Poland, using Miseq (Illumina). For genomic library preparation Nextera XT DNA sample prep kit (Illumina) was used. During machine run, paired reads of the target size 2×300 were generated. After trim procedure, 294,902 reads with at least Q30: 96.8 % were used to produce single circular contig by *de novo* assembling using Geneious 7 (Biomatters http://www.geneious.com/). The mean coverage was 156.7 with minimum 67 and maximum 904. Regions with repeated sequences were confirmed by PCR amplification and Sanger sequencing.

### Genome annotation and phylogeny

Establishment of full nucleotide sequence of DapuNPV was followed by open reading frames (ORFs) prediction. ORFs were identified only when they had been indicated by two or more following tools that use different algorithms (with exceptions when published data indicates otherwise): Glimmer3 (gene model pre-computed on OpMNPV genome) [63], GeneMarkS (parameter: Intron less eukaryotic-virus) [64] (http://linux1.softberry.com/berry.phtml?topic=virus0&group=programs&subgroup=gfindv) and tcode EMBOSS 6.5.7 [65]. Putative ORFs were then annotated by search of their translated amino acid sequence in protein database using HMMER web server (phmmer, NR large collection, scoring matrix BLOSUM62 and BLOSUM45) [66]. The scans for Pfam domains, signal peptides, disordered, transmembrane and coiled-coil regions were integrated into HMMER search and their results were presented for all previously unknown putative ORFs [40, 41, 67, 68]. For repeated sequences the Tandem Repeats Finder and PHOBOS software were used [69, 70]. Promoter motifs were searched in the 150 bp upstream region from first nucleotide of any predicted ORF. Early promoter was recognized when TATA box was present and late when DTAAG box could be found. Amino acid sequence corresponding to predicted genes was extracted and used for multiple alignment with proteins belonging to other baculoviruses. MAFFT (Multiple Alignment using Fast Fourier Transform) software with automatic algorithm selection was used. Files containing aligned sequences were exported to MEGA6 (Molecular Evolutionary Genetics Analysis) software for phylogenetic analysis [71, 72]. In order to be certain of the choice of the correct substitution model and its parameters for inferred evolutionary history, two tests that are built in MEGA6 software were performed: first based on Bayesian Information Criterion and second corrected Akaike Information Criterion. The phylogeny was established by using the Maximum Likelihood method based on the LG + G + I + F matrix model [73]. The percentage of 1000 bootstrap replicates in which the associated baculoviruses clustered together is shown next to the branches. For tree showing all sequenced genomes of baculoviruses only topology is presented. The tree showing phylogeny of *cbp* is drawn to scale, with branch lengths measured in the number of substitutions per site. The time tree shown for coding collinear genome fragments of *Alphabaculoviruses* group Ib (Fig. 5) was generated using the RelTime method [74]. Collinear genomic sequences from different baculoviruses were aligned using Mauve algorithm implemented in Geneious 7 software [75]. This method allows to recognize homologous regions of multiple sequences and align them to each other.

## Additional files

Additional file 1: Table S1. Predicted ORFs and repeat regions in the genome of DapuNPV. This file lists the ORFs predicted (with its direction and promoter sequences) in the genome of DapuNPV and their homologues in 15 other completely sequenced baculoviruses. (XLSX 31 kb) Additional file 2: Table S2. List of sequenced to date baculoviruses. This file lists all baculoviruses sequenced to date, with their accession number, genome length and source of the sequence. (DOCX 31 kb) Additional file 3: Figure S1. Alignment of OpMNPV *p8.9* ORF and its homologue in DapuNPV genome. Large insertion after nucleotide 201 in DapuNPV gene does not change the reading frame, although it decreases total basicity of translated protein which is a specific feature of p8.9 protein from OpMNPV (MAFT multiple alignment with default settings, visualization in Geneious R7). (PNG 80 kb)
