# Supplementary material for: The genome of Dasychira pudibunda nucleopolyhedrovirus (DapuNPV) reveals novel genetic connection between baculoviruses infecting moths of the Lymantriidae family
Source: BMC Genomics. 2015 Oct 8;16:759. doi: 10.1186/s12864-015-1963-9 (PMC4599791; doi:10.1186/s12864-015-1963-9)
Supplement: Additional file 2: Table S2. — List of sequenced to date baculoviruses. This file lists all baculoviruses sequenced to date, with their accession number, genome length and source of the sequence. (DOCX 31 kb) [file 12864_2015_1963_MOESM2_ESM.docx]

**Table S2 List of sequenced to date baculoviruses.**

|  | NAME | ACCESSION NUMBER (GeneBank) | Genome length (kbp) | SOURCE |
| --- | --- | --- | --- | --- |
| 1 | AcMNPV | NC_001623 | 133,894 | Ayres et al., 1994 |
| 2 | AdhoNPV | NC_004690 | 113,220 | Nakai et al., 2003 |
| 3 | AdorGV | NC_005038 | 99,657 | Wormleaton et al., 2003 |
| 4 | AdorNPV | NC_011423 | 111,724 | Hilton and Winstanley, 2008 |
| 5 | AgipMNPV | NC_011345 | 155,122 | Harrison, 2009 |
| 6 | AgNPV | NC_008520 | 132,239 | Oliveira et al., 2006 |
| 7 | AgseGV | NC_005839 | 131,680 | Ai et al., 2004* |
| 8 | AgseNPV | NC_007921 | 147,544 | Jakubowska et al., 2005* |
| 9 | AnpeNPV | NC_008035 | 126,629 | Nie et al., 2007 |
| 10 | ApciNPV | NC_018504 | 123,876 | Zhang et al., 2009* |
| 11 | BmNPV | NC_001962 | 128,413 | Gomi et al., 1999 |
| 12 | BomaNPV | NC_012672 | 126,770 | Xu et al., 2010 |
| 13 | BusuNPV | NC_023442 | 120,420 | Zhu et al., 2014 |
| 14 | CaLGV-Henan | NC_022646 | 101,818 | Liang et al., 2013 |
| 15 | CfDEFMNPV | NC_005137 | 131,160 | Lauzon et al., 2005 |
| 16 | CfMNPV | NC_004778 | 129,593 | de Jong et al., 2005 |
| 17 | ChchNPV | NC_007151 | 149,622 | van Oers et al., 2005 |
| 18 | ChmuNPV | NC_023177 | 124,688 | Rohrmann et al., 2014 |
| 19 | ChocGV | NC_008168 | 104,710 | Escasa et al., 2006 |
| 20 | ChocNPV | NC_021925 | 128,446 | Thumbi et al., 2013 |
| 21 | ChroNPV | NC_021924 | 129,052 | Thumbi et al., 2013 |
| 22 | ClanGV | NC_015398 | 101,487 | Liang et al., 2011 |
| 23 | ClbiNPV | NC_008293 | 135,454 | Zhu et al., 2009 |
| 24 | CpGV | NC_002816 | 123,500 | Luque et al., 2001 |
| 25 | CrleGV | NC_005068 | 110,907 | Lange and Jehle, 2003 |
| 26 | CuniNPV | NC_003084 | 108,252 | Afonso et al., 2001 |
| 27 | DapuNPV | KP747440 | 136,761 | this paper |
| 28 | EcobNPV | NC_008586 | 131,204 | Ma et al., 2007 |
| 29 | EpapGV | NC_018875 | 119,082 | Ferrelli et al. 2012 |
| 30 | EppoNPV | NC_003083 | 118,584 | Hyink et al., 2002 |
| 31 | ErelGV | KJ406702 | 102,759 | Ardisson-Araujo et al., 2014 |
| 32 | EupsNPV | NC_012639 | 141,291 | Tang et al., 2009 |
| 33 | HearGV | NC_010240 | 169,794 | Harrison and Popham, 2008 |
| 34 | HearMNPV | NC_011615 | 154,196 | Tang et al., 2008* |
| 35 | HearNPV | NC_003094 | 130,759 | Zhang et al., 2005 |
| 36 | HearNPV-G4 | NC_002654 | 131,405 | Chen et al., 2000* |
| 37 | HearNPV-NNg1 | NC_011354 | 132,425 | Ogembo et al., 2009 |
| 38 | HearSNPV-AC53 | NC_024688 | 130,442 | Noune and Hauxwell, 2014* |
| 39 | HespNPV | NC_021923 | 140,633 | Rohrmann et al., 2013 |
| 40 | HycuNPV | NC_007767 | 132,959 | Ikeda et al., 2006 |
| 41 | HzSNPV | NC_003349 | 130,869 | Chen et al., 2002 |
| 42 | LdMNPV | NC_001973 | 161,046 | Kuzio et al., 1999 |
| 43 | LeseNPV | NC_008348 | 168,041 | Xiao and Qi, 2007 |
| 44 | LyxyMNPV | NC_013953 | 156,344 | Nai et al., 2010 |
| 45 | MabrNPV-K1 | NC_023681 | 152,710 | Choi et al., 2013 |
| 46 | MacoNPV-A | NC_003529 | 155,060 | Li Q. et al., 2002a |
| 47 | MacoNPV-B | NC_004117 | 158,482 | Li L. et al, 2002b |
| 48 | MaviMNPV | NC_008725 | 111,953 | Chen et al., 2008 |
| 49 | NeabNPV | NC_008252 | 84,264 | Duffy et al., 2006 |
| 50 | NeleNPV | NC_005906 | 81,755 | Lauzon et al., 2004 |
| 51 | NeseNPV | NC_005905 | 86,462 | Garcia-Maruniak et al., 2004 |
| 52 | OpMNPV | NC_001875 | 131,995 | Ahrens et al., 1997 |
| 53 | OrleNPV | NC_010276 | 156,179 | Thumbi et al., 2011 |
| 54 | PealNPV-GR167 | NC_024625 | 151,109 | Rohrmann et al., 2014* |
| 55 | PhcyNPV | JX_404026 | 125,376 | Qian et al., 2013 |
| 56 | PhopGV | NC_004062 | 119,217 | Croizier et al., 2002* |
| 57 | PlxyGV | NC_002593 | 100,999 | Hashimoto et al., 2000 |
| 58 | PlxyMNPV | NC_008349 | 134,417 | Harrison and Lynn, 2007 |
| 59 | PrGV | NC_013797 | 108,592 | Zhang et al., 2012 |
| 60 | PsunGV | NC_013772 | 176,677 | Li et al., 2008a* |
| 61 | RoMNPV | NC_004323 | 131,526 | Harrison and Bonning, 2003 |
| 62 | SeMNPV | NC_002169 | 135,611 | Ijkel et al., 1999 |
| 63 | SfMNPV | NC_009011 | 131,331 | Wolff et al., 2008 |
| 64 | SpliGV | NC_009503 | 124,121 | Wang et al., 2008 |
| 65 | SpliNPV | NC_003102 | 139,342 | Pang et al., 2001 |
| 66 | SpliNPV-II | NC_011616 | 148,634 | Li et al., 2008b* |
| 67 | SujuNPV | KJ_676450 | 135,952 | Liu et al., 2014 |
| 68 | ThorNPV-p2 | NC_019945 | 132,978 | Wang et al., 2012 |
| 69 | TnSNPV | NC_007383 | 134,394 | Willis et al., 2005 |
| 70 | XcGV | NC_002331 | 178,733 | Hayakawa et al., 1999 |

- - unpublished, direct submission to NCBI database

Literature:

1. Afonso CL, Tulman ER, Lu Z, Balinsky CA, Moser BA, Becnel JJ, Rock DL, Kutish GF: **Genome sequence of a baculovirus pathogenic for *Culex nigripalpus***. *Journal of virology* 2001, **75**(22):11157-11165.
2. Ahrens CH, Russell RL, Funk CJ, Evans JT, Harwood SH, Rohrmann GF: **The sequence of the *Orgyia pseudotsugata* multinucleocapsid nuclear polyhedrosis virus genome**. *Virology* 1997, **229**(2):381-399.
3. Ai XL, Wang ZF, Wang B, Zhang W, Li F, Fu JH, Cui CS, Shi YH, He M, 2004 - *unpublished; direct submission to NCBI*
4. Ardisson-Araujo DM, de Melo FL, Andrade Mde S, Sihler W, Bao SN, Ribeiro BM, de Souza ML: **Genome sequence of *Erinnyis ello* granulovirus (ErelGV), a natural cassava hornworm pesticide and the first sequenced sphingid-infecting betabaculovirus**. *BMC genomics* 2014, **15**(1):856.
5. Ayres MD, Howard SC, Kuzio J, Lopez-Ferber M, Possee RD: **The complete DNA sequence of *Autographa californica* nuclear polyhedrosis virus**. *Virology* 1994, **202**(2):586-605.
6. Chen X, Ijkel WF, Tarchini R, Sun X, Sandbrink H, Wang H, Peters S, Zuidema D, Lankhorst RK, Vlak JM, Hu Z, 2000 – *unpublished; direct submission to NCBI*
7. Chen X, Zhang WJ, Wong J, Chun G, Lu A, McCutchen BF, Presnail JK, Herrmann R, Dolan M, Tingey S *et al*: **Comparative analysis of the complete genome sequences of *Helicoverpa zea* and *Helicoverpa armigera* single-nucleocapsid nucleopolyhedroviruses**. *The Journal of general virology* 2002, **83**(Pt 3):673-684.
8. Chen YR, Wu CY, Lee ST, Wu YJ, Lo CF, Tsai MF, Wang CH: **Genomic and host range studies of *Maruca vitrata* nucleopolyhedrovirus**. *The Journal of general virology* 2008, **89**(Pt 9):2315-2330.
9. Choi JB, Heo WI, Shin TY, Bae SM, Kim WJ, Kim JI, Kwon M, Choi JY, Je YH, Jin BR *et al*: **Complete genomic sequences and comparative analysis of *Mamestra brassicae* nucleopolyhedrovirus isolated in Korea**. *Virus genes* 2013, **47**(1):133-151.
10. Croizier L, Taha A, Croizier G, Lopez Ferber M, 2002 – *unpublished; direct submission to NCBI*
11. de Jong JG, Lauzon HA, Dominy C, Poloumienko A, Carstens EB, Arif BM, Krell PJ: **Analysis of the *Choristoneura fumiferana* nucleopolyhedrovirus genome**. *The Journal of general virology* 2005, **86**(Pt 4):929-943.
12. Duffy SP, Young AM, Morin B, Lucarotti CJ, Koop BF, Levin DB: **Sequence analysis and organization of the *Neodiprion abietis* nucleopolyhedrovirus genome**. *Journal of virology* 2006, **80**(14):6952-6963.
13. Escasa SR, Lauzon HA, Mathur AC, Krell PJ, Arif BM: **Sequence analysis of the *Choristoneura occidentalis* granulovirus genome**. *The Journal of general virology* 2006, **87**(Pt 7):1917-1933.
14. Ferrelli ML, Salvador R, Biedma ME, Berretta MF, Haase S, Sciocco-Cap A, Ghiringhelli PD, Romanowski V: **Genome of *Epinotia aporema* granulovirus (EpapGV), a polyorganotropic fast killing betabaculovirus with a novel thymidylate kinase gene**. *BMC genomics* 2012, **13**:548.
15. Garcia-Maruniak A, Maruniak JE, Zanotto PM, Doumbouya AE, Liu JC, Merritt TM, Lanoie JS: **Sequence analysis of the genome of the *Neodiprion sertifer* nucleopolyhedrovirus**. *Journal of virology* 2004, **78**(13):7036-7051.
16. Gomi S, Majima K, Maeda S: **Sequence analysis of the genome of *Bombyx mori* nucleopolyhedrovirus**. *The Journal of general virology* 1999, **80 ( Pt 5)**:1323-1337.
17. Harrison RL: **Genomic sequence analysis of the Illinois strain of the *Agrotis ipsilon* multiple nucleopolyhedrovirus**. *Virus genes* 2009, **38**(1):155-170.
18. Harrison RL, Bonning BC: **Comparative analysis of the genomes of *Rachiplusia ou* and *Autographa californica* multiple nucleopolyhedroviruses**. *The Journal of general virology* 2003, **84**(Pt 7):1827-1842.
19. Harrison RL, Lynn DE: **Genomic sequence analysis of a nucleopolyhedrovirus isolated from the diamondback moth, *Plutella xylostella***. *Virus genes* 2007, **35**(3):857-873.
20. Harrison RL, Popham HJ: **Genomic sequence analysis of a granulovirus isolated from the Old World bollworm, *Helicoverpa armigera***. *Virus genes* 2008, **36**(3):565-581.
21. Hashimoto Y, Hayakawa T, Ueno Y, Fujita T, Sano Y, Matsumoto T: **Sequence analysis of the *Plutella xylostella* granulovirus genome**. *Virology* 2000, **275**(2):358-372.
22. Hayakawa T, Ko R, Okano K, Seong SI, Goto C, Maeda S: **Sequence analysis of the *Xestia c-nigrum* granulovirus genome**. *Virology* 1999, **262**(2):277-297.
23. Hilton S, Winstanley D: **Genomic sequence and biological characterization of a nucleopolyhedrovirus isolated from the summer fruit tortrix, *Adoxophyes orana***. *The Journal of general virology* 2008, **89**(Pt 11):2898-2908.
24. Hyink O, Dellow RA, Olsen MJ, Caradoc-Davies KM, Drake K, Herniou EA, Cory JS, O'Reilly DR, Ward VK: **Whole genome analysis of the *Epiphyas postvittana* nucleopolyhedrovirus**. *The Journal of general virology* 2002, **83**(Pt 4):957-971.
25. Ijkel WF, van Strien EA, Heldens JG, Broer R, Zuidema D, Goldbach RW, Vlak JM: **Sequence and organization of the *Spodoptera exigua* multicapsid nucleopolyhedrovirus genome**. *The Journal of general virology* 1999, **80 ( Pt 12)**:3289-3304.
26. Ikeda M, Shikata M, Shirata N, Chaeychomsri S, Kobayashi M: **Gene organization and complete sequence of the *Hyphantria cunea* nucleopolyhedrovirus genome**. *The Journal of general virology* 2006, **87**(Pt 9):2549-2562.
27. Jakubowska AK, van Oers MM, Ziemnicka J, Lipa JJ, Vlak JM, 2005 - *unpublished; direct submission to NCBI*
28. Kuzio J, Pearson MN, Harwood SH, Funk CJ, Evans JT, Slavicek JM, Rohrmann GF: **Sequence and analysis of the genome of a baculovirus pathogenic for *Lymantria dispar***. *Virology* 1999, **253**(1):17-34.
29. Lange M, Jehle JA: **The genome of the *Cryptophlebia leucotreta* granulovirus**. *Virology* 2003, **317**(2):220-236.
30. Lauzon HA, Jamieson PB, Krell PJ, Arif BM: **Gene organization and sequencing of the *Choristoneura fumiferana* defective nucleopolyhedrovirus genome**. *The Journal of general virology* 2005, **86**(Pt 4):945-961.
31. Lauzon HA, Lucarotti CJ, Krell PJ, Feng Q, Retnakaran A, Arif BM: **Sequence and organization of the *Neodiprion lecontei* nucleopolyhedrovirus genome**. *Journal of virology* 2004, **78**(13):7023-7035.
32. Li L, Donly C, Li Q, Willis LG, Keddie BA, Erlandson MA, Theilmann DA: **Identification and genomic analysis of a second species of nucleopolyhedrovirus isolated from *Mamestra configurata***. *Virology* 2002, **297**(2):226-244.
33. Li Q, Donly C, Li L, Willis LG, Theilmann DA, Erlandson M: **Sequence and organization of the *Mamestra configurata* nucleopolyhedrovirus genome**. *Virology* 2002, **294**(1):106-121.
34. Li Y, Tang P, Zhang Z, Zhang H, Qin Q, 2008a - *unpublished; direct submission to NCBI*
35. Li Y, Tang P, Zhu J, Zhang Z, 2008b - *unpublished; direct submission to NCBI*
36. Liang Z, Zhang X, Yin X, Cao S, Xu F: **Genomic sequencing and analysis of *Clostera anachoreta* granulovirus**. *Archives of virology* 2011, **156**(7):1185-1198.
37. Liang Z, Zhang X, Yin X, Song X, Shao X, Wang L: **Comparative analysis of the genomes of *Clostera anastomosis* (L.) granulovirus and *Clostera anachoreta* granulovirus**. *Archives of virology* 2013, **158**(10):2109-2114.
38. Liu X, Yin F, Zhu Z, Hou D, Wang J, Zhang L, Wang M, Wang H, Hu Z, Deng F: **Genomic Sequencing and Analysis of *Sucra jujuba* Nucleopolyhedrovirus**. *PloS one* 2014, **9**(10):e110023.
39. Luque T, Finch R, Crook N, O'Reilly DR, Winstanley D: **The complete sequence of the *Cydia pomonella* granulovirus genome**. *The Journal of general virology* 2001, **82**(Pt 10):2531-2547.
40. Ma XC, Shang JY, Yang ZN, Bao YY, Xiao Q, Zhang CX: **Genome sequence and organization of a nucleopolyhedrovirus that infects the tea looper caterpillar, *Ectropis obliqua***. *Virology* 2007, **360**(1):235-246.
41. Nai YS, Wu CY, Wang TC, Chen YR, Lau WH, Lo CF, Tsai MF, Wang CH: **Genomic sequencing and analyses of *Lymantria xylina* multiple nucleopolyhedrovirus**. *BMC genomics* 2010, **11**:116.
42. Nakai M, Goto C, Kang W, Shikata M, Luque T, Kunimi Y: **Genome sequence and organization of a nucleopolyhedrovirus isolated from the smaller tea tortrix, *Adoxophyes honmai***. *Virology* 2003, **316**(1):171-183.
43. Nie ZM, Zhang ZF, Wang D, He PA, Jiang CY, Song L, Chen F, Xu J, Yang L, Yu LL *et al*: **Complete sequence and organization of *Antheraea pernyi* nucleopolyhedrovirus, a dr-rich baculovirus**. *BMC genomics* 2007, **8**:248.
44. Noune C, Hauxwell C, 2014 – *unpublished; direct submission to NCBI*
45. Ogembo JG, Caoili BL, Shikata M, Chaeychomsri S, Kobayashi M, Ikeda M: **Comparative genomic sequence analysis of novel *Helicoverpa armigera* nucleopolyhedrovirus (NPV) isolated from Kenya and three other previously sequenced Helicoverpa spp. NPVs**. *Virus genes* 2009, **39**(2):261-272.
46. Oliveira JV, Wolff JL, Garcia-Maruniak A, Ribeiro BM, de Castro ME, de Souza ML, Moscardi F, Maruniak JE, Zanotto PM: **Genome of the most widely used viral biopesticide: *Anticarsia gemmatalis* multiple nucleopolyhedrovirus**. *The Journal of general virology* 2006, **87**(Pt 11):3233-3250.
47. Pang Y, Yu J, Wang L, Hu X, Bao W, Li G, Chen C, Han H, Hu S, Yang H: **Sequence analysis of the *Spodoptera litura* multicapsid nucleopolyhedrovirus genome**. *Virology* 2001, **287**(2):391-404.
48. Qian H, Zhang Y, Wu Y, Sun P, Zhu S, Guo X, Gao K, Xu A, Wang W: **Analysis of the genomic sequence of *Philosamia cynthia* nucleopolyhedrin virus and comparison with *Antheraea pernyi* nucleopolyhedrin virus**. *BMC genomics* 2013, **14**:115.
49. Rohrmann GF, Erlandson MA, Theilmann DA: **The genome of a baculovirus isolated from *Hemileuca sp.* encodes a serpin ortholog**. *Virus genes* 2013, **47**(2):357-364.
50. Rohrmann GF, Erlandson MA, Theilmann DA: **Genome Sequence of an *Alphabaculovirus* Isolated from *Choristoneura murinana***. *Genome announcements* 2014, **2**(1).
51. Rohrmann GF, Erlandson MA, Thielmann D.A, 2014 – *unpublished; direct submission to NCBI*
52. Tang P, Li YN, Zhang H, Qin QL, Zhang ZF, 2008 - *unpublished; direct submission to NCBI*
53. Tang XD, Xiao Q, Ma XC, Zhu ZR, Zhang CX: **Morphology and genome of *Euproctis pseudoconspersa* nucleopolyhedrovirus**. *Virus genes* 2009, **38**(3):495-506.
54. Theze J, Takatsuka J, Li Z, Gallais J, Doucet D, Arif B, Nakai M, Herniou EA: **New insights into the evolution of *Entomopoxvirinae* from the complete genome sequences of four entomopoxviruses infecting *Adoxophyes honmai*, *Choristoneura biennis*, *Choristoneura rosaceana*, and *Mythimna separata***. *Journal of virology* 2013, **87**(14):7992-8003.
55. Thumbi DK, Beliveau C, Cusson M, Lapointe R, Lucarotti CJ: **Comparative genome sequence analysis of *Choristoneura occidentalis* Freeman and *C. rosaceana* Harris (Lepidoptera: Tortricidae) alphabaculoviruses**. *PloS one* 2013, **8**(7):e68968.
56. Thumbi DK, Eveleigh RJ, Lucarotti CJ, Lapointe R, Graham RI, Pavlik L, Lauzon HA, Arif BM: **Complete sequence, analysis and organization of the *Orgyia leucostigma* nucleopolyhedrovirus genome**. *Viruses* 2011, **3**(11):2301-2327.
57. van Oers MM, Abma-Henkens MH, Herniou EA, de Groot JC, Peters S, Vlak JM: **Genome sequence of *Chrysodeixis chalcites* nucleopolyhedrovirus, a baculovirus with two DNA photolyase genes**. *The Journal of general virology* 2005, **86**(Pt 7):2069-2080.
58. Wang Y, Choi JY, Roh JY, Woo SD, Jin BR, Je YH: **Molecular and phylogenetic characterization of *Spodoptera litura* granulovirus**. *Journal of microbiology* 2008, **46**(6):704-708.
59. Wang YS, Huang GH, Cheng XH, Wang X, Garretson TA, Dai LY, Zhang CX, Cheng XW: **Genome of *Thysanoplusia orichalcea* multiple nucleopolyhedrovirus lacks the superoxide dismutase gene**. *Journal of virology* 2012, **86**(21):11948-11949.
60. Willis LG, Seipp R, Stewart TM, Erlandson MA, Theilmann DA: **Sequence analysis of the complete genome of *Trichoplusia ni* single nucleopolyhedrovirus and the identification of a baculoviral photolyase gene**. *Virology* 2005, **338**(2):209-226.
61. Wolff JL, Valicente FH, Martins R, Oliveira JV, Zanotto PM: **Analysis of the genome of *Spodoptera frugiperda* nucleopolyhedrovirus (SfMNPV-19) and of the high genomic heterogeneity in group II nucleopolyhedroviruses**. *The Journal of general virology* 2008, **89**(Pt 5):1202-1211.
62. Wormleaton S, Kuzio J, Winstanley D: **The complete sequence of the *Adoxophyes orana* granulovirus genome**. *Virology* 2003, **311**(2):350-365.
63. Wormleaton S, Kuzio J, Winstanley D: **The complete sequence of the *Adoxophyes orana* granulovirus genome**. *Virology* 2003, **311**(2):350-365.
64. Xiao H, Qi Y: **Genome sequence of *Leucania seperata* nucleopolyhedrovirus**. *Virus genes* 2007, **35**(3):845-856.
65. Xu YP, Ye ZP, Niu CY, Bao YY, Wang WB, Shen WD, Zhang CX: **Comparative analysis of the genomes of *Bombyx mandarina* and *Bombyx mori* nucleopolyhedroviruses**. *Journal of microbiology* 2010, **48**(1):102-110.
66. Zhang BQ, Cheng RL, Wang XF, Zhang CX: **The Genome of *Pieris rapae* Granulovirus**. *Journal of virology* 2012, **86**(17):9544.
67. Zhang YA, Fang X, Qu LJ, Hou YX, 2009 - *unpublished; direct submission to NCBI*
68. Zhu SY, Yi JP, Shen WD, Wang LQ, He HG, Wang Y, Li B, Wang WB: **Genomic sequence, organization and characteristics of a new nucleopolyhedrovirus isolated from *Clanis bilineata* larva**. *BMC genomics* 2009, **10**:91.
69. Zhu Z, Yin F, Liu X, Hou D, Wang J, Zhang L, Arif B, Wang H, Deng F, Hu Z: **Genome sequence and analysis of *Buzura suppressaria* nucleopolyhedrovirus: a group II *Alphabaculovirus***. *PloS one* 2014, **9**(1):e86450.
