# Supplementary figures and images for: The genome of Dasychira pudibunda nucleopolyhedrovirus (DapuNPV) reveals novel genetic connection between baculoviruses infecting moths of the Lymantriidae family
Source: BMC Genomics. 2015 Oct 8;16:759. doi: 10.1186/s12864-015-1963-9 (PMC4599791; doi:10.1186/s12864-015-1963-9)

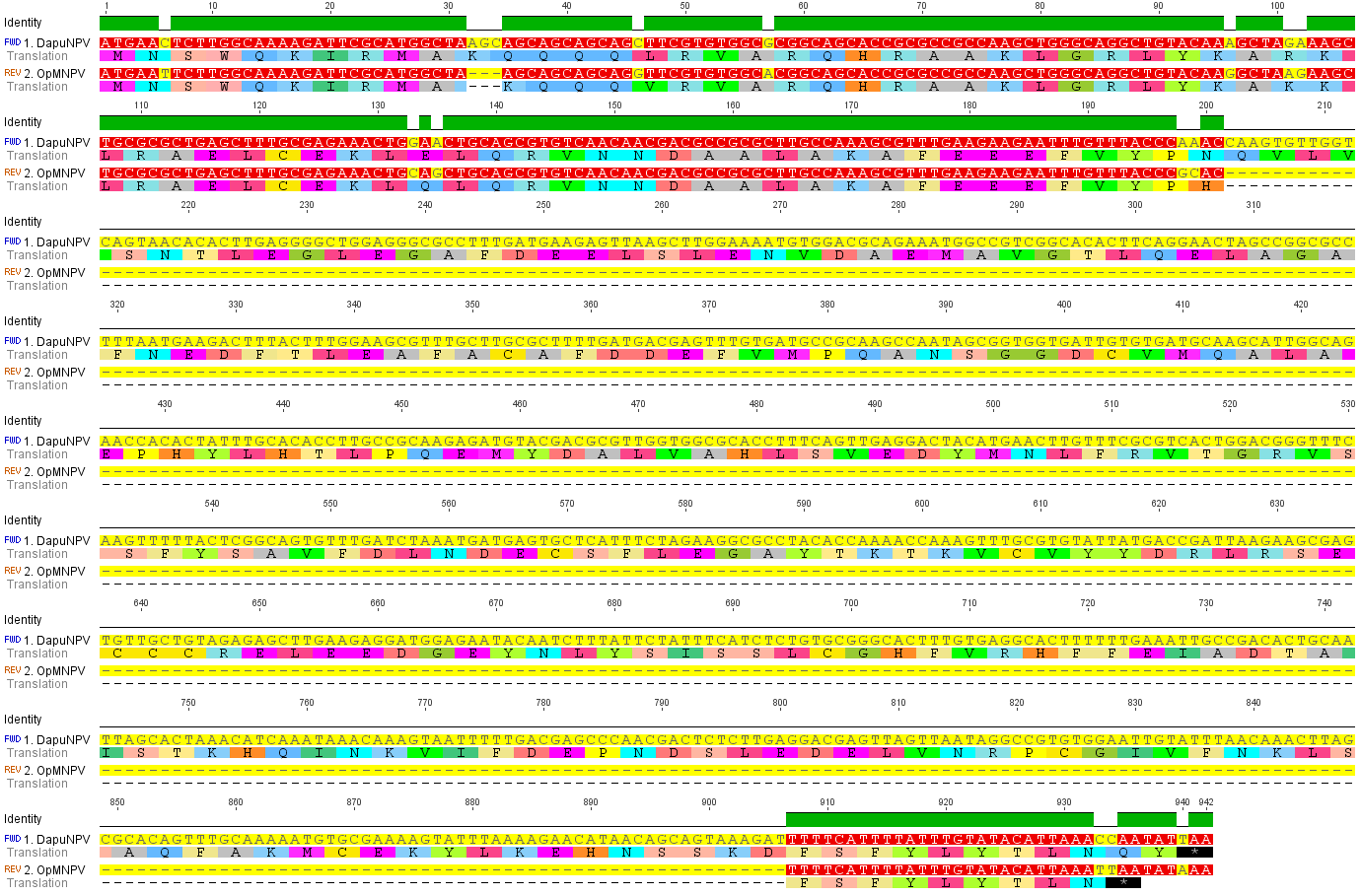

Supplement: Additional file 3: Figure S1. — Alignment of OpMNPV p8.9 ORF and its homologue in DapuNPV genome. Large insertion after nucleotide 201 in DapuNPV gene does not change the reading frame, although it decreases total basicity of translated protein which is a specific feature of p8.9 protein from OpMNPV (MAFT multiple alignment with default settings, visualization in Geneious R7). (PNG 80 kb) [file 12864_2015_1963_MOESM3_ESM.png]
